# Is Parents’ ADHD Symptomatology Associated With the Clinical Feasibility or Effectiveness of a Psychoeducational Program Targeting Their Children’s ADHD?

**DOI:** 10.1177/10870547221092120

**Published:** 2022-05-01

**Authors:** Therese Lindström, Axel Kierkegaard Suttner, Martin Forster, Sven Bölte, Tatja Hirvikoski

**Affiliations:** 1 Karolinska Institutet and Stockholm Health Care Services, Region Stockholm, Stockholm, Sweden; 2 Stockholm Health Care Services, Region Stockholm, Sweden; 3 Karolinska Institutet, Stockholm, Sweden; 4 Curtin University, Perth, WA, Australia

**Keywords:** ADHD, parental ADHD, multiplex families, psychoeducational intervention, parenting intervention

## Abstract

**Objective:**

To examine if the clinical feasibility and effectiveness of a psychoeducational program targeting children’s ADHD differ between parents with varying ADHD symptom severities.

**Method:**

An open trial analyzed data from 549 parents of children with ADHD aged 3 to 17 years, who participated in psychoeducation at an outpatient habilitation/disability clinic. In all analyses, parents were stratified into three symptom severity groups (low/middle/high) based on scores on an ADHD screening scale.

**Results:**

Parental ADHD symptom severity was not associated with results on any outcome, although the odds of having incomplete data were higher in parents reporting higher symptom severity. Across the entire sample, we observed high program completion rates, positive acceptability ratings and large increases in parental knowledge.

**Conclusions:**

Psychoeducation may be beneficial regardless of the participating parent’s ADHD symptomatology. Further research is needed to examine whether these results are generalizable to parents diagnosed with ADHD, an underrepresented group in our study.

ADHD is a neurodevelopmental disability characterized by a persistent pattern of inattention, disorganization, and/or hyperactivity-impulsivity that causes functional impairment across most areas of life (American Psychiatric Association [APA], 2013; Bölte et al., 2018). The prevalence of this highly heritable condition (Nikolas & Burt, 2010) has been estimated at 5.9% in children (Willcutt, 2012) and 2.5% to 2.8% in adults (Fayyad et al., 2017; Simon et al., 2009). Multiplex families where not only the child but also the parent(s) have ADHD symptoms, a description that applies to 20% to 40% of families of children with ADHD (Starck et al., 2016; Takeda et al., 2010), might need additional support by services or adaptations to facilitate active and beneficial participation in recommended interventions (Johnston et al., 2012). A growing number of studies indicates that parental ADHD symptoms may complicate the administration of and adherence to interventions for children’s ADHD, ultimately increasing the risk of suboptimal treatment outcomes—whether they be medication, parenting interventions (Chronis-Tuscano et al., 2017), or a combination (Rasmussen et al., 2018). Considering the familial aggregation of ADHD (Epstein et al., 2000; Uchida et al., 2021), it is important to find out if this also applies to psychoeducation, that is, whether the clinical feasibility and effectiveness of psychoeducational interventions targeting children’s ADHD differ between parents with varying ADHD symptom severities. To the best of our knowledge, this has not yet been studied.

Treatment guidelines generally recommend that the assessment of children’s ADHD be followed by structured information about the diagnosis and its treatment (Faraone et al., 2021; Taylor et al., 2004). Children are largely dependent upon their parents/caregivers to organize their treatment, and psychoeducation on children’s ADHD is often directed at their parents. Such parent-received psychoeducation typically seeks to increase parents’ knowledge about ADHD (symptoms, behavioral manifestations, impairments, etiology), and its comorbidities and treatment options. Additional objectives may include reducing unfavorable attitudes toward the diagnosed child, increasing parents’ confidence in their ability to influence their child’s situation, and providing a brief introduction to parenting strategies for managing the child’s ADHD symptoms and behaviors (Dahl et al., 2020).

The accessibility of an intervention has implications for its clinical feasibility, including completion rates and program acceptability. Psychoeducation is most often delivered in a standardized educational group format, where didactic lectures form a central part (Dahl et al., 2020). Psychoeducational programs designed for adults with ADHD can yield significant improvements in knowledge about ADHD (Hirvikoski et al., 2017). Participation does, nevertheless, place high demands on cognitive capacities that are often challenged in adults with ADHD; including inhibitory control, working memory and selective/sustained attention, as well as the ability to memorize, sort, and prioritize provided information. Relatedly, it has been suggested that adult ADHD symptoms may interfere with participation in parenting interventions, for example by causing difficulties in organizing treatment participation and assimilating program content (Chronis-Tuscano et al., 2017; Johnston et al., 2012). Parents in multiplex families may also, by virtue of their own ADHD symptomatology, be at increased risk for a range of psychosocial factors associated with poorer treatment attendance and premature treatment termination (Nock & Ferriter, 2005), including socioeconomic disadvantage (Erskine et al., 2016), co-occurring psychiatric conditions such as depression (Minde et al., 2003), and a history of dropouts from educational or occupational engagements (Soendergaard et al., 2016).

When it comes to the effects of parent-received psychoeducation, its positive role has been supported across several trials (Dahl et al., 2020; Montoya et al., 2011). The findings are, however, neither easy to interpret nor consistent, as the format, scope, and outcomes of the interventions vary considerably between studies (Dahl et al., 2020). Given common conceptualizations of psychoeducation as a base and catalyst for the continued ADHD treatment process, it is surprising that few studies include changes in knowledge about ADHD among their outcomes (Bai et al., 2015; Dahl et al., 2020; Schoenfelder et al., 2020). Indeed, parental ADHD knowledge seems to be of importance not only for parents’ attitudes toward ADHD (Amiri et al., 2016), and available treatment options (Corkum et al., 1999), but also for their causal attributions about symptoms and behaviors displayed by their ADHD child (Johnston & Freeman, 2002). In line with calls to consider parents’ prerequisites, needs, and levels of parental stress when intervening with (their) children’s ADHD (Chacko et al., 2017; Theule et al., 2013), it is important to find out how parents with high ADHD symptom severity participate in and benefit from psychoeducation targeting their children’s ADHD.

The objective of this study was to examine whether the clinical feasibility (in terms of completion rates and acceptability) and effectiveness of a parent-received psychoeducational program on children’s ADHD differ between parents with varying ADHD symptom severities. The program under study is widely implemented across Sweden, with good clinical feasibility (Bölte et al., 2020; Svanborg et al., 2009). However, little is known about how it works for parents who themselves have ADHD symptoms. Overall, it was hypothesized that parents with high ADHD symptom severity would have lower program completion rates (primary feasibility outcome) and benefit less than parents with low ADHD symptom severity, for example in terms of parental knowledge gains (primary effectiveness outcome).

## Method

### Clinical Setting

This open trial was performed at the ADHD Center, Habilitation & Health, Region Stockholm, Sweden. The ADHD Center is a publicly-funded outpatient habilitation service clinic that offers courses, workshops, and lectures on ADHD for families of children and adolescents with ADHD, aged 3 to 17 years. Throughout the Stockholm region, families of children who have been diagnosed with ADHD following procedures for a formal ADHD assessment (National care program ADHD, 2019) are recommended to enroll themselves at the ADHD Center and its psychoeducational introductory courses on ADHD.

The project was approved by the Regional Ethics Committee in Stockholm (2017/575-31/5). Participants received information about the study and its procedures in written and oral format before giving their written consent.

### Participants and Procedure

**Figure 1.**
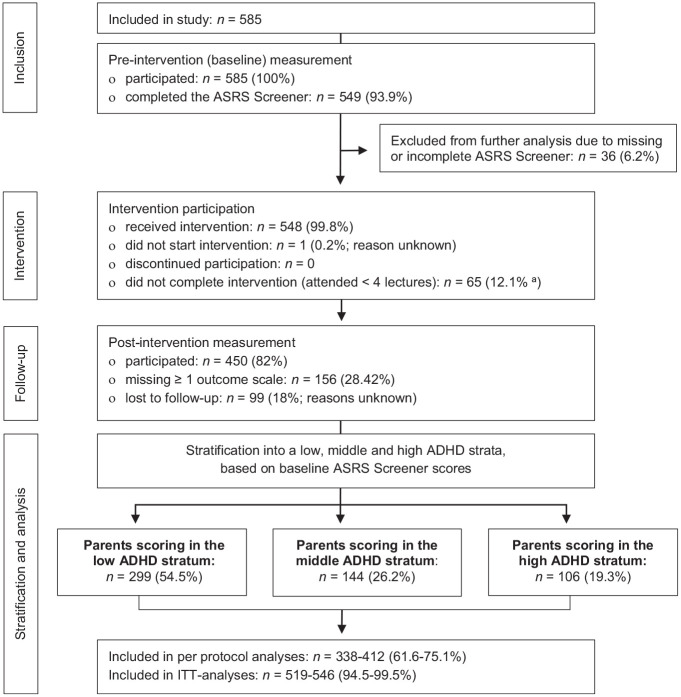
Flow chart of participants included and analyzed in this uncontrolled psychoeducation trial. *Note*. ASRS = Adult ADHD Self-Report Scale; ITT = intention to treat. ^a^Information on attendance missing for *n* = 13 (2.4%), why percentage was calculated as proportion of *n* = 536.

A total of 585 parents of children and adolescents with ADHD aged 3 to 17 years were recruited from among families enrolling in the psychoeducational program under study at the ADHD Center. Of these, the 549 participants (93.9%) that had a complete baseline rating on the Adult ADHD Self-Report Scale (ASRS) Screener were included in further analyses (Figure 1). Of the 36 parents (6.2%) who did not have a complete ASRS Screener, 4 had missed single items while 32 (88.9%) had not filled in the scale at all (reasons unknown). The ADHD diagnosis of the child for whom the parent enrolled in psychoeducation (hereafter referred to as the “target child”) was confirmed in accordance with the ADHD Center’s clinical routines, by psychologists with access to the child’s medical health records and reports. The study had no explicit exclusion criteria. However, parents with a known intellectual disability and parents who were judged to urgently need other kinds of treatment or support (e.g., due to a severe or acute psychiatric condition) were referred to appropriate health care units and interventions in accordance with the ADHD Center’s regular clinical procedures. Parents of the same child were allowed to participate in the program and the study at the same time.

The recruitment period lasted between May 2017 and November 2019. The recruitment procedure was further adapted at two occasions, due to clinical routine changes at the ADHD Center and a low initial participation rate. Initially, all parents who signed up for the program under study were invited to also participate in its evaluation (n = 183, 33.3% of the study participants). Later, some (of several parallel) program rounds were dedicated to the evaluation in such a way that all parents who signed up for these particular course rounds also participated in the study (n = 366, 66.7%). Parents recruited before and after the routine changes did not differ in regard to their baseline ASRS Screener scores (p = .18).

### Intervention

The structured psychoeducational program under study is developed for parents of children (4–12 years) and adolescents (13–17 years) with ADHD. An earlier version of the program received positive evaluations in a trial of ADHD pharmacotherapy delivered in combination with psychoeducation (Svanborg et al., 2009) and the clinical feasibility of the current version of the program has been supported in a survey of regional ADHD care quality (Bölte et al., 2020). It was originally designed to be accessible and helpful also for parents who themselves have ADHD symptoms. The ADHD Center has since devoted substantial effort into further improving their program delivery approach and use of inclusive practices (see Table 1 for examples).

**Table 1.** Examples of General ADHD Adaptations to Promote Program Accessibility and Facilitate Active Participation in the Psychoeducational Program Under Study.

Overall approach	The design and the delivery of the program is informed and permeated by: • an ambition to provide psychoeducation in an inclusive and accessible way • a recognition that circumstances, challenges, and needs vary between families • an awareness that some of the participants may have ADHD symptoms and related needs, regardless of whether this is known in advance • the assumption that “what is helpful for parents with ADHD, is probably helpful for most parents.” • Lecturers are encouraged to: • respond to parents and their experiences in a warm, open, accepting, and validating way • use a non-stigmatizing language, adapted to the terms and wording used by the parents • instruct parents to use the provided information in ways that make sense in and fit with the context of their own family’s current situation and needs.
Lecture structure	The lectures are highly structured, frequently alternating between short/condensed informative lectures and interactive elements. Lecturers provide carefully selected information in a clear, concise, and simple way. Information is processed and concretized through structured group discussions, work sheets, and role play-based illustrations by lecturers. Lecturers use cognitive and visual aids, such as time-timers, slides/pictures, and large note pads. Parents are encouraged to do what they themselves need to enable focus and active participation (e.g., stand up, take breaks, use strategies of their own). Extra energy (e.g., coffee, fruit, candy) is provided during frequently occurring breaks.
Other	Lecturers are encouraged to: • attend to any need for support in getting heard, as well as in regulating or restricting oneself • greet parents who arrive late with a warm welcome • encourage parents to catch up on missed lectures.

The program consists of five 3-hour lectures providing information about ADHD, treatment options and societal support services, as well as environmental modifications and parenting strategies that may facilitate the everyday management of the target child’s needs and behaviors (details in Supplemental Table 1). Lectures are held for groups of 25 to 35 parents, guided by a detailed manual and slide presentation. At the ADHD Center, each lecture is delivered by a clinical psychologist. A total of 15 psychologists with program-specific training were involved in the current trial.

Treatment integrity was, due to practical circumstances, rated only in the final year of data collection, during which 182 parents (33.2%) participated and 42.5% of lectures were observed. Across the monitored lectures, all (100%) of the manualized content was delivered during 94.1% of the occasions (allowing postponement of some content from one lecture to the following in three cases). In addition, all (100%) criteria related to program structure adherence and provision of material were fulfilled during 82.4% of the observed lectures. The treatment integrity ratings were made by a research assistant (psychology student), using standardized checklists.

### Data Collection and Measures

Participants completed a questionnaire covering a range of sociodemographic factors and rated their own overall well-being on a scale of 1 (*worst possible*) to 10 (*best possible*). The program’s clinical feasibility was evaluated based on program completion rates and ratings of program acceptability. The program’s effectiveness was examined by self-report questionnaires, completed at baseline (pre) and immediately after (post) the intervention.

#### Parental ADHD symptoms

The Adult ADHD Self-Report Scale (ASRS) is an ADHD screening scale designed to measure the frequency of recent adult ADHD symptoms (Kessler et al., 2005). The short six-item ASRS Screener used in the current study has been found to outperform the full-length scale when it comes to distinguishing between clinical and non-clinical ADHD cases, with a total classification accuracy of 97.9% and an area under the curve value of 0.84 (Kessler et al., 2005). Respondents are asked to state how often a particular ADHD symptom has occurred during the past 6 months on a 5-point Likert-type scale from 0 (*never*) to 4 (*very often*). Responses are then dichotomized, yielding a score between 0 and 6. To further enhance the usefulness of the ASRS Screener in reproducing clinical ADHD evaluations, scores are collapsed into three strata: low (corresponding to scores 0–1), middle (scores 2–3), and high (scores 4–6). Using these strata, Kessler et al. (2005) found that 68.7% of clinical ADHD cases scored in the high stratum (and thus at a level equivalent to a positive ADHD screening), while no more than 4.3% scored in the low. In contrast, no more than 0.5% of non-clinical cases scored in the high stratum, while 74.8% scored in the low. The validity of the ASRS Screener has also been found adequate in a Swedish general population sample (Lundin et al., 2019). In the sample of the current study, the internal consistency (as estimated by Cronbach’s alpha) of the ASRS Screener was *r*α = .81.

#### Clinical feasibility outcomes

**Completion rate:** Attendance was documented continuously. Parents attending at least four (i.e., 80%) of the five lectures were considered to have completed the program.

**Acceptability ratings:** Lecture acceptability and satisfaction was evaluated with a treatment evaluation scale (Bramham et al., 2009; Hirvikoski et al., 2017) modified to fit the program under study and to match the ADHD Center’s regular evaluation forms. After each lecture, participants rated their agreement with four statements scored on a scale 0 (*not at all*) to 3 (*yes absolutely*). Three items targeted lecture contents (e.g., relevance and usefulness) and a fourth addressed the experience of in-lecture discussions. A summary lecture evaluation score was calculated by averaging each respondent’s ratings across items, with higher mean scores (ranging between 0 and 3) taken to indicate a more positive appraisal. Overall program satisfaction was evaluated with an extended version of these lecture evaluation forms, completed after the program. In addition to items addressing the overall content relevance, usefulness, and in-lecture sharing of experiences, the scale was supplemented with a summary grade (mimicking school grades *fail*, *pass*, *pass with distinction*, and *pass with special distinction*) and a question of whether the participant would recommend the program to other parents. Once again, items were rated on a scale ranging from 0 to 3, and a higher mean score (ranging between 0 and 3) was taken to indicate a more positive appraisal of the program. Cronbach’s alpha was *rα* = .80 for the overall program satisfaction scale and varied between *rα* = .74 and *rα* = .81 for the five lecture satisfaction scales.

#### Effectiveness outcomes

**Knowledge quiz:** The participants’ knowledge about ADHD, treatment options, societal services, and parenting strategies (i.e., topics covered by the program under study) was measured with a modified version of an ADHD quiz (Bramham et al., 2009). This 20-item quiz consisted of 17 statements (e.g., *Children with ADHD also have deficient executive functions*) scored on a *true*-, *false*-, or don’t *know*-scale and three short-answer questions (e.g., *What percentage of Swedish school-aged children have an ADHD diagnosis*?). Each correct answer was assigned one credit, yielding a total sum score between 0 and 20. Higher scores were taken to indicate more knowledge. When administered in the current sample, the Knowledge Quiz was found to have good discriminant properties, reaching a post intervention average item-difficulty of p = .75 (min p = .29, max p = .96), thus meeting the ≥ .75 threshold often set for false/true tests (Cohen & Swerdlik, 2005). The internal consistency (as estimated with the Kuder-Richardson 20 [KR_20_] formula, a dichotomous equivalent to Cronbach’s alpha) of the Knowledge Quiz was KR_20_ = .63.

**Strengths and Difficulties Questionnaire (SDQ):** The general behavioral symptoms and attributes of the participants’ target children were measured with the extended version of the SDQ for parents of 4 to 17 year-olds (Goodman, 1997, 1999). The SDQ Total Difficulties Scale consists of 20 items covering emotional symptoms, hyperactivity/inattention, conduct problems, and peer relationship problems. Responses are marked as *not true*, *somewhat true*, or *certainly true* (scored 0, 1, and 2, respectively) and can be summed to yield a total score between 0 and 40. The SDQ also comprises a five-item prosocial behaviors subscale, with sum scores ranging from 0 to 10. Moreover, the SDQ has an impact supplement that asks parents to rate the extent to which their child’s difficulties cause distress or social impairment: *not at all*, *only a little*, *quite a lot*, or *a great deal* (scored 0, 0, 1, and 2, respectively). When summed, this SDQ Impact Score ranges from 0 to 10. Overall, higher scores indicate more difficulties (Total Difficulties Scale), more prosocial behaviors (Prosocial Subscale), or more everyday impairment (Impact Score). In the current sample, Cronbach’s alpha was *rα* = .68 for the Total Difficulties Scale, *rα* = .73 for the Prosocial Subscale, and *rα* = .72 for the Impact Score.

**The Parental Stress Scale (PSS):** The participants’ levels of parental stress were measured with the PSS (Berry & Jones, 1995). The scale consists of 18 statements (e.g., *I feel overwhelmed by the responsibility of being a parent*) rated on a 5-point Likert-type scale from 1 (*strongly disagree*) to 5 (*strongly agree*). Responses are summed to yield a total PSS score between 18 and 90. Higher scores indicate higher levels of parental stress. In the current sample, Cronbach’s alpha was *rα* = .84.

**Parental attributions (PA):** The participants’ causal attributions about undesired behaviors displayed by their target child were measured with a modified version of the Written Analog Questionnaire (Johnston & Freeman, 1997; Johnston & Ohan, 2005). The scale was altered to fit parents of children with varying ages (3–17) and ADHD in different presentations, both pre- and post-intervention. First, parents were asked to state which behavior of their target child they currently perceived as the most troublesome (e.g., *does not listen; forgets things; gets outbursts, reacts unexpectedly strongly; other*). Next, parents were asked to think about a situation where the stated behavior occurred in a typical manner and rate their assumptions about its causes on six 10-point scales from 1 to 10, addressing the following: the causal locus, the stability and the globality of the behavior, the child’s degree of control over the behavior, the intentionality of the behavior, and the parent’s responsibility for the occurrence of the behavior. Then, parents were asked to do the very same thing once again, while rating the assumed causes for the second most troublesome behavior of their target child. In line with previously published procedures (Johnston & Freeman, 1997; Johnston & Ohan, 2005), we combined the ratings of globality and stability into a single score and averaged ratings across the firstly and the secondly scored behaviors, yielding a single set of five PA dimensions 1 to 10. After some reverse-scoring, higher scores were taken to indicate an attribution of the child’s behavior as being *more* dispositional (internal or stable/global), controllable or intentional, and of one’s own parental responsibility for the occurrence of the behavior as being *lower.* In the current sample, Spearman Brown split-half coefficients were estimated to .50 for one of the two-item PA dimensions (Controllability) and seen to vary between .62 and .67 for the others (PA Stability/Globality, PA Locus, PA Intentionality, and PA Responsibility [Eisinga et al., 2013]).

#### Missing data

The extent of missing data was substantial post intervention, which is why we post hoc chose to analyze the potential associations between data incompleteness and parental ADHD symptom severity. Scales missing ≤10% of item scores were treated as complete after item-level imputation: missing Knowledge Quiz items were replaced with a value (credit) of 0, while missing SDQ and PSS items were replaced with the average of the respondent’s observed items. Pairwise exclusion was used to handle scales missing >10% of items, as well as missing sociodemographic data.

### Statistical Analysis

Based on the assumption that adults with a positive ADHD screening are more likely to experience impairment, we wanted to differentiate between parents who reported high ADHD symptom severity (screening positively for ADHD) and parents who did not. Accordingly, participants were stratified into groups corresponding to the low, middle, and high strata defined by Kessler et al. (2005). For each ADHD stratum, observed data was screened for accuracy, completeness, and fits with assumptions of planned analyses. There were generally few outliers, and there were no extreme values.

The odds of completing the program were examined in a binary logistic regression, using the ADHD strata variable as predictor. A series of one-way ANOVAs were conducted to examine whether parents scoring in the low, middle, or high ADHD strata differed in their ratings of lecture or program evaluations, or in terms of baseline scores on the effectiveness-related measures. Significant effects were further investigated using Tukey’s post hoc tests.

The effectiveness-related outcomes were analyzed in a series of mixed ANOVAs conducted with *time* (pre, post) as within-subjects factor and *group* (low, middle, high ADHD strata) as between-subjects factor. Due to the significant amount of missing data, the primary analyses were performed per protocol (including only complete cases). They were then repeated according to the intention-to-treat (ITT) principle (including program completers as well as non-completers with pre-intervention data), with missing values replaced using the last observation carried forward procedure. For the sake of brevity, results from these secondary ITT analyses are reported in detail only when they differ significantly from results obtained per protocol. The same applies to the supplementary analyses in which parents’ and children’s baseline medication status were included as covariates. Cohen’s *d* was interpreted as 0.20 = small, 0.50 = medium, and 0.80 = large (Cohen, 1988). Partial eta squared (*n*_p_^2^) was interpreted as .01 = small, .06 = moderate, and .14 = large (Cohen, 1988).

Finally, three binary logistic regression analyses were performed to examine if the odds of having incomplete data differed between parents in the low, middle, and high ADHD strata. Statistical analyses were performed in IBM SPSS Statistics, version 26. Cohen’s *d* was calculated and one of the figures was drawn in RStudio, version 1.4.1106.

## Results

### Parental ADHD

Of the 549 parents who had a complete ASRS Screener, 299 (54.5%) scored in the low ADHD stratum, 144 (26.2%) scored in the middle ADHD stratum, and 106 (19.3%) scored in the high ADHD stratum (i.e., at a level equivalent to a positive ADHD screening). Among the 22 parents (4%) who reported that they currently had an ADHD diagnosis, 2 (9.1%) scored in the low ADHD stratum, 4 (18.2%) scored in the middle, and 16 (72.7%) scored in the high.

### Demographic Data and Baseline Comparisons

Parents in the low, middle, and high ADHD strata differed in terms of educational levels, proportion working full-time, and ratings of their own well-being (Table 2), but not in regard to characteristics of their target child (Table 3). Specifically, the proportion of parents working full-time was smaller in the high ADHD stratum than in the low (p < .001). The proportion of parents with neither post-secondary nor secondary education was larger in the high ADHD stratum than in the low (p < .001). Parents in the high ADHD stratum reported lower overall well-being than parents in the low (p = .002).

**Table 2.** Baseline Characteristics of the Participants, Summarized for the Total Sample as Well as for Parents Scoring in the Low, Middle, and High ADHD Strata Separately.

	All (*n* = 549)		Low ADHD stratum (*n* = 299)		Middle ADHD stratum (*n* = 144)		High ADHD stratum (*n* = 106)		Strata comparisons
	*M* (*SD*)	Min–max	*M* (*SD*)	Min–max	*M* (*SD*)	Min–max	*M* (*SD*)	Min–max	*p-*Value^a^
Age	43.34 (6.63)	26–73	43.67 (6.95)	29–73	43.43 (6.16)	30–62	42.32 (6.27)	26–63	.20
ASRS screener score	1.71 (1.73)	0–6	0.34 (0.48)	0–1	2.48 (0.50)	2–3	4.54 (0.69)	4–6	.00**
Well-being (1-10)	6.56 (2.02)	1–10	6.79 (2.08)	2–10	6.48 (1.82)	2-10	5.99 (2.04)	1–10	.00* High < low**
	*n*	%	*n*	%	*n*	%	*n*	%	
ADHD diagnosis (current)	22	4.01	2	0.67	4	2.78	16	15.09	.00**
Female gender	334	60.84	195	65.22	80	55.56	59	55.66	.06
Highest education									.00*
Upper secondary	229	41.71	116	38.80	69	47.92	44	41.51	
University	273	49.73	165	55.18	64	44.44	44	41.51	
Other	36	6.56	13	4.35	8	5.56	15	14.15	High > low**
Occupation									.00 **
Work full time	407	74.13	237	79.26	110	76.39	60	56.60	High < low**
Work part time	71	12.93	36	12.04	14	9.72	21	19.81	
Other	60	10.93	21	7.02	17	11.81	22	20.75	
Pharmacotherapy^b^	91	16.58	43	14.38	24	16.67	24	22.64	.08
Non-pharmacologicalintervention^c^	59	10.75	34	11.37	11	7.64	14	13.21	.33
Full time w/ target child	416	75.77	220	73.58	109	75.69	87	82.08	.19
Two adults at home^d^	436	79.42	233	77.93	112	77.78	91	85.85	.11
Partner has ADHD or ADHD symptoms	131	23.86	77	25.75	30	20.83	24	22.64	.41
Other parent/caregiver participate in the program	334	60.84	192	64.21	81	56.25	61	57.55	.53
Other parent/caregiver participate in the study	188	34.24	114	38.13	42	29.17	32	30.19	.34

*Note*. Percentages are calculated as proportion of total sample. Values are missing for 0-18 individuals (0-3.3%). ASRS = Adult ADHD Self-Report Scale; w/ = with.

a From one-way ANOVAs (continuous variables), Chi-square tests or Fischer’s exact tests (categorical variables).

b Medication to treat ADHD or to improve mental health (current).

c Psychological or psychosocial intervention (current).

d Both biological parents (*n* = 365, 66.5%) or participant and partner/step parent (*n* = 71, 12.9%) live together.

* Significant at an <.05 level, **significant at an <.001 level.

**Table 3.** Baseline Characteristics of the Participants’ Target Children, Summarized for the Total Sample as Well as for Parents Scoring in the Low, Middle, and High ADHD Strata Separately.

Age	All (*n* = 549)		Low ADHD stratum (*n* = 299)		Middle ADHD stratum (*n* = 144)		High ADHD stratum (*n* = 106)		Strata comparisons
	*M (SD)*	Min–max	*M (SD)*	Min–max	*M (SD)*	Min–max	*M (SD)*	Min–max	*p*-Value^a^
	10.45 (2.85)	3-17	10.62 (2.79)	3-17	10.54 (2.92)	4-17	9.88 (2.88)	5-17	.07
	*n*	%	*n*	%	*n*	%	*n*	%	
Female gender	164	29.87	91	30.43	41	28.47	32	30.19	.76
ADHD (any form)^b^	549	100.00	299	100.00	144	100.00	106	100.00	.69
ADHD combined	328	59.74	176	58.86	90	62.50	62	58.49	
ADHD inattentive	108	19.67	59	19.73	28	19.44	21	19.81	
ADHD hyperactive-impulsive	38	6.92	21	7.02	7	4.86	10	9.43	
ADHD other	57	10.38	34	11.37	16	11.11	7	6.60	
ADHD medication	258	46.99	146	48.83	69	47.92	43	40.57	.33
≥One parallel contact^c^	245	44.63	138	46.15	63	43.75	44	41.51	.69

*Note*. Percentages are calculated as proportion of total sample. Values are missing for 0 to 10 individuals (0%–1.8%).

a From one-way ANOVAs (continuous variables) or Chi-square tests (categorical variables).

b Twelve children (2.2%) also had an autism spectrum disorder, in addition to their ADHD.

c At least one additional contact, for example, within the child and adolescent (primary or secondary) psychiatric care.

At baseline, parents in the high ADHD stratum reported higher parental stress than parents in the low (PSS, d = 0.52 [95% CI = 0.25, 0.78], p = .001; Table 4). The same pattern was seen for ratings of the everyday impact of the behavioral symptoms of the participants’ target children (SDQ Impact Score, Table 4), although post hoc tests revealed no statistically significant differences (all p ≥ .07). Parents in the three ADHD strata did not differ in terms of baseline knowledge measured with the Knowledge Quiz (p = .68; Table 4).

**Table 4.** Results From a Series of Mixed ANOVAs Calculated Per Protocol From Pre- to Post-Intervention, Presented Along With Baseline Differences.

	*n*	Pre			Post			Baseline differences (between the ADHD strata)	Overall effect of intervention (main effect of time)		Interaction effect	
		Low ADHD stratum	Middle ADHD stratum	High ADHD stratum	Low ADHD stratum	Middle ADHD stratum	High ADHD stratum					
		*M* (*SD*)	*M* (*SD*)	*M* (*SD*)	*M* (*SD*)	*M* (*SD*)	*M* (*SD*)	*F*(*df*), *p*	*F*(*df*), *p*	Partial *n*^2^, *d* (95% CI)	*F*(*df*)	Partial*n*^2^
Knowledge Quiz	412	9.67 (2.96)	9.55 (3.34)	9.96 (3.67)	15.12 (2.32)	14.73 (2.64)	14.89 (2.39)	F(2,409) = 0.39, *p* = .68	F(1,409) = 1,050.87, p = .00**	*n_p_*^2^ = .72, d = 1.83 (1.67, 1.99)	F(2,409) = 1.03, p = .36	*n_p_*^2^ = .01
PSS	388	39.31 (8.93)	41.74 (10.52)	43.97 (9.36)	39.33 (8.71)	40.74 (10.13)	42.19 (9.21)	F(2,385) = 7.48, p = .00*	F(1,385) = 7.92, p = .01*	*n_p_*^2^ = .02, d = −0.06 (−0.12, −0.00)	F(2,385) = 3.04, p = .05*	*n_p_*^2^ = .02
SDQ Total Difficulties	392	17.23 (5.14)	18.15 (5.16)	18.62 (4.71)	16.42 (5.13)	17.47 (4.90)	17.95 (4.93)	F(2,389) = 2.58, p = .08	F(1,389) = 13.62, p = .00**	*n_p_*^2^ = .03, d = −0.15 (−0.22, −0.08)	F(2,389) = 0.07, *p* = .93	*n_p_*^2^ = .000
SDQ Prosocial Subscale	392	7.16 (2.11)	7.15 (2.13)	6.66 (2.44)	7.38 (2.10)	7.23 (2.00)	7.05 (2.20)	F(2,389) = 1.56, p = .21	F(1,389) = 7.97, p = .01*	*n_p_*^2^ = .02, d = −0.10 (−0.17, −0.03)	F(2,389) = 0.91, p = .40	*n_p_*^2^ = .005
SDQ Impact Score	338	4.03 (2.12)	4.68 (2.29)	4.72 (2.47)	3.23 (2.14)	3.90 (2.64)	4.11 (2.42)	F(2,335) = 3.77, p = .02*	F(1,335) = 39.40, p = .00**	*n_p_*^2^ = .11, d = −0.33 (−0.42, −0.24)	F(2,335) = 0.27, *p* = .77	*n_p_*^2^ = .002
PA Stability/Globality	391	8.33 (1.32)	8.34 (1.29)	8.66 (1.12)	8.11 (1.31)	7.97 (1.51)	8.25 (1.32)	F(2,388) = 1.98, p = .14	F(1,388) = 16.70, p = .00**	*n_p_*^2^ = .04, d = −0.22 (−0.33, −0.11)	F(2,388) = 0.65, *p* = .52	*n_p_*^2^ = .003
PA Intentionality	381	4.29 (2.45)	4.25 (2.25)	4.25 (2.32)	3.47 (2.31)	3.42 (1.93)	3.51 (2.32)	F(2,378) = 0.01, p = .99	F(1,378) = 37.20, p = .00**	*n_p_*^2^ = .09, d = −0.35 (−0.45, −0.25)	F(2,378) = 0.04, *p* = .96	*n_p_*^2^ = .000
PA Locus	378	6.57 (2.13)	6.25 (2.16)	6.34 (2.07)	6.12 (2.30)	5.91 (2.22)	6.01 (2.32)	F(2,375) = 0.82, p = .44	F(1,375) = 5.93, p = .02*	*n_p_*^2^ = .02, d = −0.18 (−0.30, −0.06)	F(2,375) = 0.08, *p* = .92	*n_p_*^2^ = .000
PA Controllability	387	3.99 (1.89)	4.27 (2.01)	3.83 (1.89)	3.72 (1.96)	3.46 (1.64)	3.65 (2.00)	F(2,384) = 1.18, p = .31	F(1,384) = 13.03, p = .00**	*n_p_*^2^ = .03, d = −0.20 (−0.31, −0.09)	F(2,384) = 2.65, *p* = .07	*n_p_*^2^ = .01
PA Responsibility	388	6.94 (2.22)	6.83 (1.89)	6.70 (2.23)	6.57 (2.39)	6.61 (1.92)	6.13 (2.34)	F(2,385) = 0.37, p = .69	F(1,385) = 9.62, p = .00*	*n_p_*^2^ = .02, d = −0.17 (−0.27, −0.07)	F(2,385) = 0.53, *p* = .59	*n_p_*^2^ = .003

*Note*. Knowledge Quiz = measure of parental knowledge about ADHD, treatment options, and parenting strategies; PA = Parental Attributions, measure of parents’ causal attributions about their child’s behaviors; PSS = Parental Stress Scale, measure of parental stress; SDQ = Strengths and Difficulties Questionnaire, measure of the general behavioral symptoms and attributes of the participant’s target child.

* Significant at an <.05 level, **significant at an <.001 level.

At baseline, 16.6% of the parents had pharmacotherapy of their own, to treat ADHD or to improve mental health (Table 2). Post intervention, 10.4% of participants (12% in the low, 28.5% in the middle, and 8.5% in the high ADHD stratum) reported that their target child had started pharmacological ADHD treatment during the study period. Likewise, 1.3% of the parents (0.7% in the low, 0.7% in the middle, and 3.8% in the high ADHD strata) reported that they had started pharmacotherapy of their own.

### Clinical feasibility

#### Completion rate

The overall program completion rate was good, with 471 participants (87.9%) attending at least four (i.e., 80%) of the five lectures (M = 4.4, SD = 1.0; n = 13 with missing data excluded). For parents scoring in the low, middle, and high ADHD strata, the completion rates were 90%, 84.3%, and 86.8%, respectively. Using parents in the low ADHD stratum as reference, the odds of completing the program were not significantly lower in the middle (OR = 0.60 [95% CI = 0.33, 1.08], p = .09) or in the high ADHD stratum (OR = 0.73 [95% CI = 0.37, 1.44], p = .37). There were no significant differences between program completers and non-completers in terms of ASRS Screener scores (p = .24) or when compared across a range of demographic variables such as age, gender, education, main occupation, child age, and child gender (p = .13 to .81).

#### Acceptability ratings

Overall, lecture evaluations were positive, averaging 2.7 on a 0 to 3 satisfaction scale (SD = 0.4). Ratings of the five lectures varied between M = 2.6 (SD = 0.4) and M = 2.7 (SD = 0.4; n = 404–479). Ratings of overall program acceptability averaged 2.7 on a 0 to 3 scale (SD = 0.3, n = 437). Almost all parents stated that they would probably or absolutely recommend the program under study to other parents (99.8%) and gave it a final summary grade equivalent to a school grade of *pass with distinction* or higher (95.5%). There were no statistically significant differences between parents in the three ADHD strata, neither in terms of lecture acceptability ratings (p = .15 to .81), nor in terms of program satisfaction (p = .60).

### Effectiveness

#### Parental knowledge

**Figure 2.**
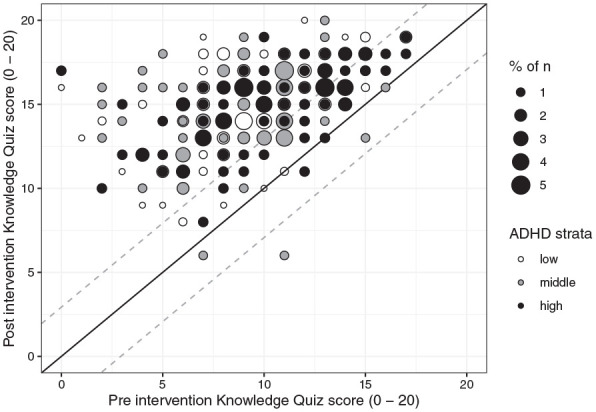
Pre- and post-intervention sum scores on the Knowledge Quiz for parents in the low, middle, and high ADHD strata who were included in per protocol analyses (n = 412). *Note.* Baseline scores are plotted along the horizontal axis, and post-intervention scores along the vertical. Participants falling on the diagonal reference line had the same score pre- and post-intervention (did not change). Participants above the reference line had higher scores post-intervention, while participants falling below the reference line had lower.

When analyzed per protocol, there was a significant main effect of time, revealing a large increase in parental knowledge from pre to post intervention (Knowledge Quiz; Figure 2, Table 4). There were no differences in the levels of knowledge gained by parents in the low, middle, and high ADHD strata (Table 4). Similar results were found in the ITT analysis: large increases in knowledge (F[1, 537] = 688.65, d = 1.29 [95% CI = 1.18, 1.41], n_p_^2^ = .56, p < .001) and no between-strata differences (F[2, 537] = 2.59, n_p_^2^ = .01, p = .08). The results did not change when parent and child medication status were included as covariates.

#### Child behaviors, parental stress, and parental attributions

When analyzed per protocol, parents reported small but statistically significant changes (reductions) in the behavioral and symptomatic difficulties of their target child (SDQ), their own levels of parental stress (PSS), and their causal attributions about undesired behaviors displayed by their target child (PA; Table 4). The only significant interaction effect was seen on the PSS (Table 4), for which pairwise comparisons of estimated marginal means revealed a small but statistically significant score reduction for parents in the high ADHD stratum (mean difference = −1.8 PSS scores [95% CI = −3.1, −0.5], p = .01), but not for parents in the low or middle strata. Similar results were obtained in ITT analyses (n = 519–546), with one exception: the interaction effect seen for the PSS was no longer statistically significant (F[2, 540] = 2.50, n_p_^2^ = .01, p = .08). The results did not change when parent and child medication status were included as covariates, with two exceptions: the small pre-to-post change on the PA Responsibility dimension did not survive (p = .08) and the interaction effect for the PA Controllability dimension got significant (p = .046; although it did not remain so in an ITT analysis including the same covariates, p = .09).

### Data completeness

The amount of missing data varied between variables but was generally smaller in the low ADHD stratum than in the middle and high strata (details in Supplemental Table 2). The average number of lecture evaluation forms missing for parents in the low, middle, and high ADHD strata was 0.9 (SD = 1.2), 1.2 (SD = 1.4), and 1.2 (SD = 1.3) out of five, respectively. The proportion of participants lost to post-intervention measurements was 16.1% in the low, 22.2% in the middle, and 17.9% in the high ADHD strata. The average number of effectiveness-related post-intervention measures missing for parents in the low, middle, and high ADHD strata were 0.82 (SD = 1.5), 1.1 (SD = 1.7), and 1.0 (SD = 1.6) out of four, respectively. The proportion of participants that missed at least one post-intervention measure was 25.1% in the low, 33.3% in the middle, and 31.1% in the high ADHD strata.

Using parents in the low ADHD stratum as references, post hoc analyses revealed that the odds of missing at least one lecture evaluation form were higher among parents in the high ADHD stratum (OR = 1.62 [95% CI = 1.02, 2.56], p = .04). Likewise, the odds of missing at least one effectiveness-related outcome measure were higher in the middle (OR = 1.61 [95% CI = 1.06, 2.45], p = .03) and in the high ADHD stratum (OR = 1.58 [95% CI = 0.99, 2.52], p = .056), although only the first result was statistically significant (Supplemental Table 3).

## Discussion

To the best of our knowledge, this is the first study to examine whether a parent-received psychoeducational program on children’s ADHD is as feasible and effective for parents with high ADHD symptom severity as it is for parents with low ADHD symptom severity. Across the total sample, this open trial revealed high program completion rates, high program acceptability ratings and large increases in parental knowledge about ADHD, its treatment, and parenting strategies. In addition, we observed small positive changes in parents’ ratings of the everyday impact of their target child’s behavioral symptoms, their levels of parental stress and their causal attributions for their offspring’s behaviors. We did not, however, detect any of the expected differences between parents with varying ADHD symptom severities — either in program completion rates or regarding knowledge gains. Rather, the results preliminarily indicated that most participants did benefit from the program under study also in the context of parental ADHD symptoms. These results are encouraging, as they suggest that parent-received psychoeducation delivered with general adaptations to promote program accessibility for adults with ADHD may indeed have the potential to be helpful for a large proportion of the families concerned. It should be noted though, that the sample’s average ADHD symptom severity levels were well below the clinical range and that the proportion of parents with a diagnosed disability in the form of ADHD (4%) was much smaller than the expected 20% to 40% (Starck et al., 2016; Takeda et al., 2010).

Looking first across the entire sample, the results regarding the clinical feasibility and the informative utility of the psychoeducational program under study were promising. When administered in its current outpatient habilitation services context, including a range of inclusive practices to promote accessibility, no less than 88% of the participants completed the program. Also in support of the program’s clinical feasibility, the parents’ evaluations of its acceptability were noticeably positive. In terms of program effectiveness, the findings were consistent with previous observations of improvements in parental knowledge following psychoeducation (Bai et al., 2015; Schoenfelder et al., 2020). In contrast to previous studies (Dahl et al., 2020), we also observed reductions in the parents’ ratings of their target child’s behavioral symptoms, as well as in their levels of parental stress. Although small, these reductions were observed after no more than 5 weeks of psychoeducation, indicating that more long-term follow-up measurements would have been of interest. Indeed, the search for and coordination of the various health care interventions and services that children with ADHD are entitled to do constitute a major, time-consuming stressor in many Swedish families (Renhorn et al., 2019). Theoretically, the psychoeducational provision of clear information about treatment options and available support may contribute to alleviating some of the pressure. Moreover, we observed small changes across a range of parental causal attribution dimensions that may have implications for parental responses to undesired behaviors on behalf of their child (Johnston & Ohan, 2005) as well as their likelihood of attending parent training interventions (Chacko et al., 2017). Although preliminary, these findings do suggest that the possibility of influencing parental causal attributions is indeed worth further investigation (Johnston & Ohan, 2005).

Contrary to what was hypothesized, the clinical feasibility and effectiveness of the program under study did not differ between parents with varying ADHD symptom severities. Instead, parents with high ADHD symptom severity were found to be as likely to complete the program, submit positive program acceptability ratings, and display knowledge gains as parents with low ADHD symptom severity. The main findings of this study thus differ from previous research linking parental ADHD symptoms and executive dysfunction to parenting- and treatment-related difficulties (Johnston et al., 2012; Park et al., 2017; Rasmussen et al., 2018), including less beneficial outcomes after other types of parenting interventions (Chronis-Tuscano et al., 2017).

First, based on these promising results, it seems that the overall accessibility of the program under study may indeed be sufficient for a large proportion of parents enrolling in psychoeducation at the ADHD Center. However, it remains unclear whether these results would apply more generally, to psychoeducation delivered with fewer adult ADHD adaptations or to a sample with other demographic characteristics.

Second, it may be that parental ADHD symptoms do not influence psychoeducation outcomes in a way that is comparable to how they seem to attenuate the effects of, for example, parent training interventions (Chronis-Tuscano et al., 2017). For example, while psychoeducation primarily provides information, parent training explicitly asks parents to also implement and adhere to the newly acquired skills and strategies at home. Indeed, Friedman et al. (2020) recently found parental ADHD symptoms to be associated with poorer skill utilization between parent training sessions, but not with other treatment engagement aspects such as skill understanding, session attendance, or treatment attitudes. The authors accordingly suggested that parental ADHD symptoms have “the greatest effect on behaviors that parents must self-initiate,” including for example skill-use (Friedman et al., 2020). Thus, future studies should investigate the potential influence of parental ADHD symptoms on what would ideally follow after psychoeducation, that is, parent-initiated translation of the newly acquired knowledge into environmental modifications, use of new parenting strategies and attempts to find, coordinate, and adhere to recommended interventions and services (Chronis-Tuscano et al., 2017; Johnston et al., 2012; Rasmussen et al., 2018).

Third, as is often the case in clinical research (Nock & Ferriter, 2005; Smith et al., 2015), we may have failed to reach and include a representative proportion of families with more complex needs, who experience more treatment barriers. Indeed, parents in the high ADHD stratum did report lower education, employment, and well-being, as well as higher parental stress than parents in the low ADHD stratum (Faraone et al., 2021; Theule et al., 2011). However, given the familial clustering of ADHD (Epstein et al., 2000; Nikolas & Burt, 2010), we had expected the sample to encompass a significantly larger proportion of parents diagnosed with ADHD than the observed 4%. Certainly, the proportion of participants who reported high ADHD symptom severity (19.3%) was larger than that previously observed in a Swedish general population sample (6.8% [Lundin et al., 2019]). However, an inspection of the proportion of participants in the high ADHD stratum that had a post-secondary education (42%) indicates that this group of parents was relatively less impaired than, for example, a sample of adults with newly-diagnosed ADHD identified in Swedish nationwide health registers (Garcia-Argibay et al., 2021) and a clinical sample of adults in psychoeducation for adults with ADHD (Hirvikoski et al., 2017). Thus, despite performing this study at a regular outpatient habilitation service center in which the program under study was a routine intervention, we may very well have included too few parents with ADHD at clinically significant and impairing levels to detect a possible association between parental ADHD symptomatology and psychoeducation outcomes. Clinical experience suggests that parents who themselves have disabilities such as ADHD are less likely to even enroll at care centers and interventions (Bölte et al., 2020). Probably, continued work is needed to improve accessibility and generate intrinsic motivation for participation.

### Limitations

The results of this uncontrolled study should be read in the light of the above-discussed limitations in sample representativeness, which do have consequences for their generalizability to parents with diagnosed ADHD. Furthermore, it should be noted that the amount of missing data was unevenly distributed in such a way that a disproportionally large percentage of parents with higher ADHD symptom severity had to be omitted from many analyses. This pattern of missingness is not surprising given drop out and data incompleteness rates in other clinical trials where adults with ADHD symptomatology participate in interventions directed to themselves (Soendergaard et al., 2016) or targeting their child’s ADHD (Rasmussen et al., 2018). Nevertheless, it meant that we had to run the primary effectiveness-related analyses per protocol. To enable supplementary ITT analyses, we used the last observation carried forward procedure to replace missing values. This technique is associated with a certain risk of bias but was judged to be the least problematic given the observed pattern of missingness and the fact that this was a pre-post study with only two measurement points, which caused problems using, for example, linear mixed models to handle missing data. In addition, all outcomes except completion rates were based on parental self-reports. Our ability to detect potential between-strata differences in program acceptability may have been reduced by ceiling effects, preventing discrimination between parents clustering at high/positive evaluation scores. Additionally, parental ADHD symptom severity was measured and defined solely based on the ASRS Screener (Kessler et al., 2005). However, similar approaches have been used successfully by most prior studies examining associations between parental ADHD symptomatology and parenting intervention outcomes (Chronis-Tuscano et al., 2017). Finally, we had no information about other or additional psychiatric condition(s) on behalf of the participants, which is why this could not be accounted for in the analyses. The same applies to information about which parents that participated in the program and/or the study on behalf of the same target child, whose ratings cannot be assumed fully independent.

## Conclusions

Our results indicate that parents with varying degrees of ADHD symptomatology, including those with high symptom severity, may indeed benefit from parent-received psychoeducation delivered with general adaptations to promote program accessibility for adults with ADHD. Further research is needed to examine whether these results can be generalized to parents with diagnosed ADHD and significant impairments in everyday functioning, whose ADHD symptom burdens are higher than those observed among parents in the current study.

## Supplemental Material

sj-pdf-1-jad-10.1177_10870547221092120 – Supplemental material for Is Parents’ ADHD Symptomatology Associated With the Clinical Feasibility or Effectiveness of a Psychoeducational Program Targeting Their Children’s ADHD? Click here for additional data file. Supplemental material, sj-pdf-1-jad-10.1177_10870547221092120 for Is Parents’ ADHD Symptomatology Associated With the Clinical Feasibility or Effectiveness of a Psychoeducational Program Targeting Their Children’s ADHD? by Therese Lindström, Axel Kierkegaard Suttner, Martin Forster, Sven Bölte and Tatja Hirvikoski in Journal of Attention Disorders
