# Supplementary material for: Is Parents’ ADHD Symptomatology Associated With the Clinical Feasibility or Effectiveness of a Psychoeducational Program Targeting Their Children’s ADHD?
Source: J Atten Disord. 2022 May 1;26(12):1653–67. doi: 10.1177/10870547221092120 (PMC9373197; doi:10.1177/10870547221092120)
Supplement: sj-pdf-1-jad-10.1177_10870547221092120 – Supplemental material for Is Parents’ ADHD Symptomatology Associated With the Clinical Feasibility or Effectiveness of a Psychoeducational Program Targeting Their Children’s ADHD? [file sj-pdf-1-jad-10.1177_10870547221092120.pdf]

Supplemental Material/Appendices

for “Is parents’ ADHD symptomatology associated with the clinical feasibility or effectiveness of a psychoeducation program targeting their children’s ADHD?”

by Therese Lindström, Axel Kierkegaard Suttner, Martin Forster,

Sven Bölte, and Tatja Hirvikoski

Including:

Supplementary Table 1

Supplementary Table 2

Supplementary Table 3

Supplementary Table 1

Focus of the Five Psychoeducational Lectures: Themes, Contents, Discussion Topics and Strategies or Tasks Parents are Encouraged to Try

	Theme and content of lecture	Topics for group discussion (examples)	Voluntary homework
**Lecture 1**	*Good to know about ADHD*. Provides basic facts about ADHD, including the DSM definition, core symptoms and manifestations, gender differences and major hypothesis on causes of ADHD. Presents information about diagnostic neuropsychiatric assessments and recommended treatments options.	▪ What are my expectations for the program?<br>▪ What ADHD symptoms and traits do I recognize in my child?<br>▪ What experiences does my family have of pharmacological treatment of ADHD?	Track how the ADHD core symptoms are expressed in your child's everyday life.
**Lecture 2**	*ADHD in the family*. Gives information on common maladaptive parent-child interaction patterns and their potential consequences, e.g., in terms of family stress. Other themes include: ADHD and peer relationships; the child's own understanding of the disability; the situation of siblings; the social environment's support - or lack thereof; lifestyle factors such as diet, physical activity and screen time, as well as their respective connections to ADHD.	▪ How do I interrupt "vicious cycles" of escalating conflict with my child?<br>▪ How have I discussed ADHD with my child?<br>▪ What are my child's strengths or interests (some examples)? What are my strengths as a parent?<br>▪ What is a recovering activity to me? What do I need, to be able to perform that activity?	If possible, take some time for a recovery activity of your own.
**Lecture 3**	*Help the child in its everyday functioning*. Presents a basic introduction to psychological and behavioral learning theories. Introduces a number of behavioral parenting strategies to facilitate the management of the ADHD child's needs and behaviors, some of which are illustrated by lecturers in roleplay. Concretizes the introduced strategies and their potential, as well as practical usefulness through group discussions.	▪ When do I have "undemanding" moments together with my child?<br>▪ When do I use positive reinforcement with my child?<br>▪ How can I make prompts and instructions to my child shorter and clearer?	Choose one parenting strategy and consciously apply it in interaction with your child.
**Lecture 4**	*Prevent and manage conflicts*. Introduces additional behavioral parenting strategies, some of which are illustrated by lecturers in roleplay. Concretizes the introduced strategies and their potential, practical usefulness through group discussions.	▪ How does my child's ADHD influence my family's most common subjects of conflict?<br>▪ What rules are functional/effective in our family?<br>▪ Which approaches or techniques help me to "keep calm"?	Choose one parenting strategy from lecture 3 or 4, and consciously apply it in interaction with your child.
**Lecture 5**	*Support services.* Presents information on Swedish laws regarding societal support services for families of children with ADHD. Provides information on academic/school-based support, financial support, municipal social services, and the health care services. Asks parents to write a letter to themselves, summarizing their personal take-home messages, which strategies they want to continue to use and what will be their "next step", going forward.	▪ What adaptations and support measures have been helpful for my child at school?<br>▪ How do we handle homework in my family?<br>▪ What are my take-home messages from the program?	-

Note. DSM = Diagnostic and Statistical Manual of Mental Disorders.

Supplementary Table 2

Summary of Missing Data Across Outcome Measures, Summarized for the Total Sample As Well As for Parents Scoring in the Low, Middle, and High ADHD Strata Separately

	**All (*n* = 549)**	**Low ADHD Stratum (*n* = 299)**	**Middle ADHD Stratum (*n* = 144)**	**High ADHD Stratum (*n* = 106)**
	n (%)	*n* (%)	*n* (%)	*n* (%)
≥ one lecture evaluation form missing	312 (56.83)	157 (52.51)	87 (60.42)	68 (64.15)
Course evaluation missing	112 (20.40)	55 (18.39)	37 (25.69)	20 (18.87)
Pre-intervention scales				
Knowledge Quiz missing	9 (1.64)	4 (1.34)	2 (1.39)	3 (2.83)
PSS missing	6 (1.09)	5 (1.67)	0 (0.00)	1 (0.94)
SDQ missing	3 (0.55)	2 (0.67)	0 (0.00)	1 (0.94)
≥ one PA dimension missing	32 (5.83)	11 (3.68)	14 (9.72)	7 (6.60)
≥ one pre scale missing	41 (7.47)	17 (5.69)	15 (10.42)	9 (8.49)
Post-intervention scales				
Knowledge Quiz missing	106 (19.31)	51 (17.06)	34 (23.61)	21 (19.81)
PSS missing	131 (23.86)	62 (20.74)	41 (28.47)	28 (26.42)
SDQ missing	129 (23.50)	64 (21.40)	39 (27.08)	26 (24.53)
≥ one PA dimension missing	144 (26.23)	69 (23.08)	46 (31.94)	29 (27.36)
≥ one post scale missing	156 (28.42)	75 (25.08)	48 (33.33)	33 (31.13)
≥ one pre and/or post scale missing	178 (32.42)	83 (27.76)	55 (38.19)	40 (37.74)

Note. PA: Parental Attributions; PSS = Parental Stress Scale; SDQ = Strengths and Difficulties Questionnaire

Supplementary Table 3

Odds of Missing Data, as Calculated by Logistic Regressions Using ADHD Strata (Low, Middle, High; Low as Reference) as Predictor

	**Low ADHD Stratum (*n* = 299)**		**Middle ADHD Stratum (*n* = 144)**					**High ADHD Stratum (*n* = 106)**				
	n (%)	Wald X^2 *(df)*	*n* (%)	Wald X^2 (df)	*OR*	*95 % CI OR*	*p*	*n* (%)	Wald X^2 (df)	*OR*	*95 % CI OR*	*p*
Lecture evaluations: miss ≥ one form	158 (52.84)	5.32 (2)	87 (60.42)	2.45 (1)	1.38	0.92, 2.07	.118	68 (64.15)	4.26 (1)	1.62	1.02, 2.56	.039 *
Course evaluation: miss course evaluation	55 (18.39)	3.35 (2)	37 (25.69)	3.12 (1)	1.53	0.95, 2.47	.077	20 (18.87)	0.01 (1)	1.03	0.58, 1.82	.914
Pre and/or post scales: miss ≥ one measure	83 (27.76)	6.48 (2)	55 (38.19)	4.90 (1)	1.61	1.06, 2.45	.027 *	40 (37.74)	3.65 (1)	1.58	0.99, 2.52	.056

Note. OR = Odds ratio (Exp(B)); 95 % CI OR = 95 % confidence interval, surrounding OR estimates

* Significant at an < .05 level
